# CT imaging-based machine learning model: a potential modality for predicting low-risk and high-risk groups of thymoma: “Impact of surgical modality choice”

**DOI:** 10.1186/s12957-021-02259-6

**Published:** 2021-05-11

**Authors:** Ayten Kayi Cangir, Kaan Orhan, Yusuf Kahya, Hilal Özakıncı, Betül Bahar Kazak, Buse Mine Konuk Balcı, Duru Karasoy, Çağlar Uzun

**Affiliations:** 1 Department of Thoracic Surgery, İbn-i Sina Hospital, Ankara University Faculty of Medicine, 06100, Sıhhiye, Ankara, Turkey; 2 Ankara University Medical Design Application and Research Center (MEDITAM), 06100, Sıhhiye, Ankara, Turkey; 3 Department of Dentomaxillofacial Radiology, Ankara University Faculty of Dentistry, 06560, Yenimahalle, Ankara, Turkey; 4 Department of Pathology, Ankara University Faculty of Medicine, 06100, Sıhhiye, Ankara, Turkey; 5 Department of Statistics, Hacettepe University Faculty of Science, 06800, Beytepe, Ankara, Turkey; 6 Department of Radiology, Ankara University Faculty of Medicine, 06100, Sıhhiye, Ankara, Turkey

**Keywords:** Radiomics, Machine learning, Thymoma, Minimally invasive surgery, Diagnostic tool

## Abstract

**Introduction:**

Radiomics methods are used to analyze various medical images, including computed tomography (CT), magnetic resonance, and positron emission tomography to provide information regarding the diagnosis, patient outcome, tumor phenotype, and the gene-protein signatures of various diseases. In low-risk group, complete surgical resection is typically sufficient, whereas in high-risk thymoma, adjuvant therapy is usually required. Therefore, it is important to distinguish between both.

This study evaluated the CT radiomics features of thymomas to discriminate between low- and high-risk thymoma groups.

**Materials and methods:**

In total, 83 patients with thymoma were included in this study between 2004 and 2019. We used the Radcloud platform (Huiying Medical Technology Co., Ltd.) to manage the imaging and clinical data and perform the radiomics statistical analysis. The training and validation datasets were separated by a random method with a ratio of 2:8 and 502 random seeds. The histopathological diagnosis was noted from the pathology report.

**Results:**

Four machine-learning radiomics features were identified to differentiate a low-risk thymoma group from a high-risk thymoma group. The radiomics feature names were Energy, Zone Entropy, Long Run Low Gray Level Emphasis, and Large Dependence Low Gray Level Emphasis.

**Conclusions:**

The results demonstrated that a machine-learning model and a multilayer perceptron classifier analysis can be used on CT images to predict low- and high-risk thymomas. This combination could be a useful preoperative method to determine the surgical approach for thymoma.

## Introduction

Radiomics is a rapidly growing field of mapping digital medical images to quantitative data, with the end goal of generating imaging biomarkers as clinical decision-making support tools. The fundamental premise of radiomics is that radiological images contain biological, prognostic, and predictive knowledge that is not revealed during visual inspection; thus, converting medical radiological images into high-dimensional data and the subsequent quantitative analysis of these data support decision-making guidelines used in clinical practice [1–3]. Radiomics is intended to predict patient-specific results based on high throughput analysis and mining of sophisticated imaging biomarkers through machine-learning algorithms.

Thymic epithelial tumors (TETs) originate from the thymus and consist of thymomas, thymic carcinomas, and thymic neuroendocrine tumors. Although rare (1.5 cases/million), thymomas are common primary tumors of the anterior mediastinum in adults. Thymic carcinomas are very rare. Thymic carcinomas often present with metastasis and involve a poorer prognosis compared to thymomas, so TETs are a heterogeneous group [4, 5]. Despite the good survival rate of patients with thymoma, the histological subtype affects tumor behavior and the prognosis. Thymomas are subdivided into types A, AB, B1, B2, and B3 according to the World Health Organization (WHO) classification. Thymomas can also be subdivided depending on the prognosis into low-risk (types A, AB, and B1) and high-risk (types B2 and B3) groups. The high-risk group of thymomas is more likely to invade locally than the low-risk group. Surgery is the main strategy to treat thymomas, and complete resection provides the best survival rate, but the thymoma subgroups are an important factor in determining the treatment approach. The possibility of complete surgical resection is very high in the low-risk group, and this is typically an adequate treatment without adjuvant or neoadjuvant chemotherapy. In contrast, the high-risk group of thymomas has less of an opportunity for complete surgical resection than the low-risk group and may require multimodal therapy. Histological classification can inform risk stratification for patients and personalize the surgical treatment course [6–10].

Robotic-assisted thoracoscopic surgery and video-assisted thoracoscopic surgery are considered minimally invasive surgeries (MISs) and have recently become common for many surgeries, including surgery for thymic neoplasms. Some studies have reported that the results obtained from MIS are equivalent to the median sternotomy approach in the surgical treatment of thymoma. However, the indication for MIS in thymoma surgery is controversial. Many surgeons, particularly those with open surgery experience, are reluctant to perform MIS to treat TETs because MIS techniques are associated with increased manipulation of the tumor during surgery and a corresponding risk for capsular disruption, pleural seeding with tumor fragments, and incomplete resection, particularly for thymic carcinomas and high-risk thymomas. Thus, MIS may cause higher local recurrence rates and lead to lower overall survival rates. The possibility of local recurrence and spread is less likely for low-risk thymomas, so MIS is an acceptable surgical treatment approach for these cases [11]. Knowing whether a thymoma is at high or low risk preoperatively would help inform the choice of surgical approach.

Contrast-enhanced chest computed tomography (CT) is the most common imaging modality to preoperatively assess thymomas. The value of qualitative CT features in determining thymic carcinoma versus thymoma or low- versus high-risk remains unclear [12, 13]. Also, both CT and magnetic resonance imaging (MRI) have limited value for predicting the histologic subtype of thymoma [14, 15]. Preoperative prediction of the histological subtype of thymoma may facilitate patient management. A pre-surgical needle biopsy is a reliable method for diagnosing thymoma, but a small biopsy sample may not always represent the entire tumor; a deep biopsy is an invasive procedure with a risk of complications, and a transpleural biopsy may cause tumor seeding [16, 17]. Also, CT-guided biopsy and a histopathological evaluation of specimens are expensive, destroy tissues, and take 10–14 days. Overall, no clear non-invasive preoperative criteria have been defined to help surgeons choose either an open thymectomy or a minimally invasive approach. Therefore, an effective and objective surgical approach to preoperatively determine the thymoma subtype would be useful.

This study analyzed the textural features of thymomas using CT radiomics features to discriminate low- versus high-risk thymoma groups in a single center.

## Materials and methods

The study protocol was approved by the Institutional Review Board of Ankara University, Faculty of Medicine (IRB no: I7-426-20). The need for informed consent was waived because the study had a retrospective design. The initial study population included 221 consecutive patients who underwent surgical resection or biopsy between 2004 and 2019 in the Thoracic Surgery Department and were diagnosed with a thymic epithelial tumor in the Department of Pathology of Ankara University Medical Faculty. Of these patients, 158 were diagnosed with thymoma. The following inclusion criteria were applied: (1) histopathologically confirmed thymoma; (2) CT images since 2011, which was the beginning of the Ankara University Faculty of Medicine electronic database archive; (3) contrast-enhanced CT performed within 4 weeks before surgery or biopsy; (4) no history of resection for a thymic neoplasm or another malignant tumor; and (5) no history of chemotherapy or radiotherapy before the primary thoracic malignancy. After applying these selection criteria, 83 patients were included (Table 1).

**Table 1:** Clinical characteristics of the patients with the low-risk group and high-risk group

Patients (*n* = 83)	Low-risk group, *n* (%) 51 (61)	High-risk group, *n* (%) 32 (39)	*p*-value*
Sex, *n* (%)			
Male	25 (49)	20 (62)	0.23
Female	26 (51)	12 (38)	
Age, median (range) (years)	50 (21–73)	50 (24–66)	0.68
Smoking status, *n* (%)			
Never	34 (67)	18 (56)	0.34
Current or past smoker	17 (33)	14 (44)	
Previous malignancy or synchronous tumor, *n* (%)			
Absent	48 (94)	27 (52)	0.25
Present	3 (6)	5 (48)	
Clinical presentation, *n* (%)			
Asymptomatic	16 (31)	10 (31)	0.99
Symptomatic	35 (69)	22 (69)	
Myasthenia gravis, *n* (%)			
Absent	43 (84)	22 (69)	0.09
Present	8 (16)	10 (31)	
LDH, median (range) (U/L)	180 (113–338)	179 (89–310)	0.80
ALP, median (range) (U/L)	68 (29–124)	67 (31–167)	0.74
CRP, median (range) (mg/L)	4.1 (0.6–70.7)	3.5 (0.1–25.8)	0.67
HGB, median (range) (g/dL)	14 (9–16.6)	13.6 (9–16.3)	0.62
WBC count, median (range) (×10^9^/L)	7.7 (3.3–16.8)	7.3 (3.9–13.9)	0.97
LYMP count, median (range) (×10^9^/L)	2.1 (0.01–6.1)	1.8 (0.5–3.8)	0.10
PLT count, median (range) (×10^9^/L)	258 (121–454)	233 (122–404)	0.58
Type of treatment, *n* (%)			
Surgery±adjuvant treatment	44 (86)	28 (87)	1.00
Definitive chemotherapy±radiotherapy	7 (14)	4 (13)	
Largest dimension of tumor size on CT (mean ± SD) (range) in mm	66.8± 2.1 (21–160)	63±2.2 (20–134)	0.55

*LDH* lactate dehydrogenase, *ALP* alkaline phosphatase, *CRP* C-reactive protein, *HGB* hemoglobin, *WBC* white blood cell, *LYMP* lmyphocyte, *PLT* platelet

*Differences were compared using the *t*-test/Mann-Whitney *U* test, Pearson Chi-square test/Fisher’s exact test

### CT protocol and lesion segmentation

**Fig. 1:**
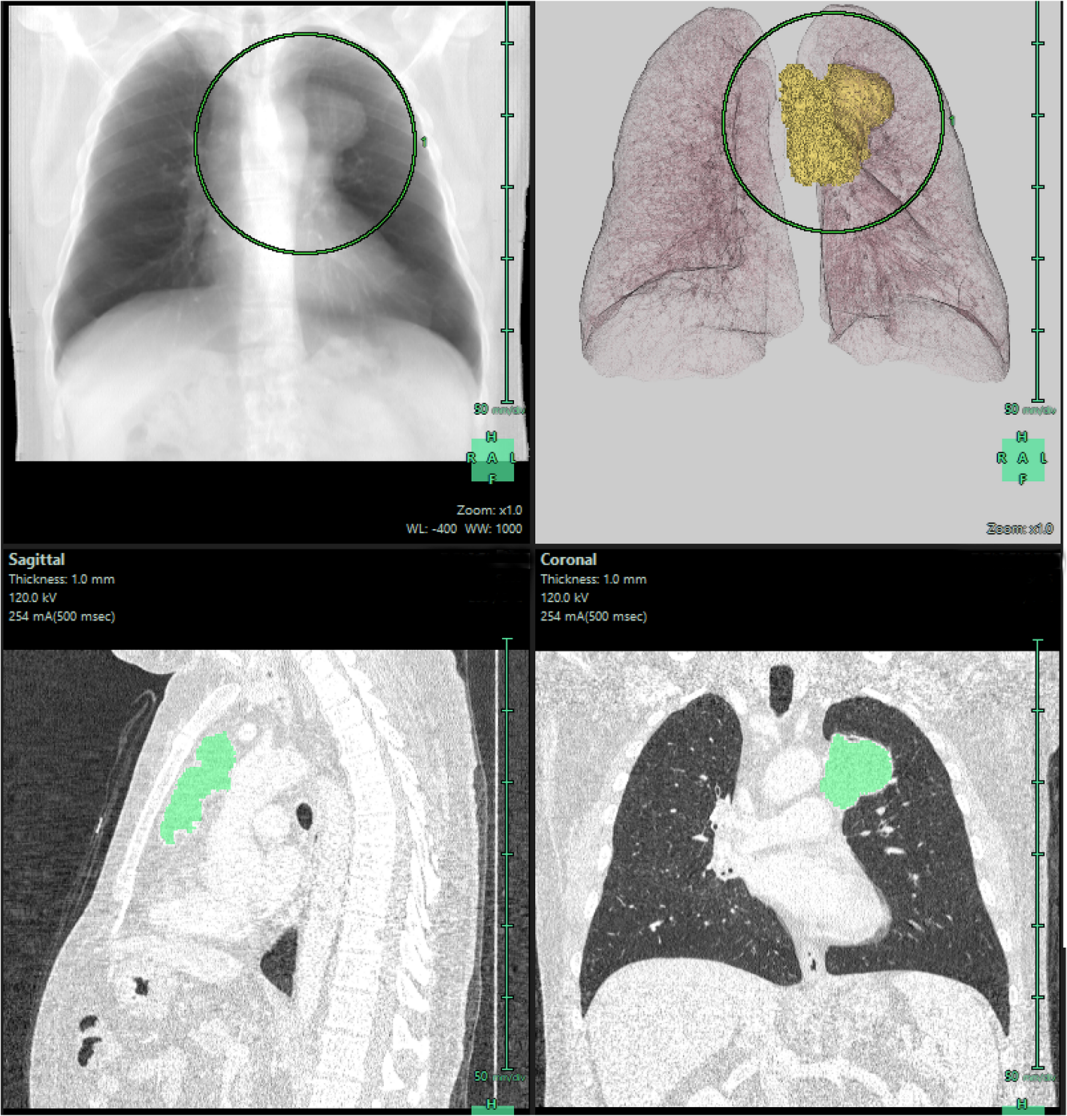
The lung extraction and 3D representation of tumor with lung structures

**Fig. 2:**
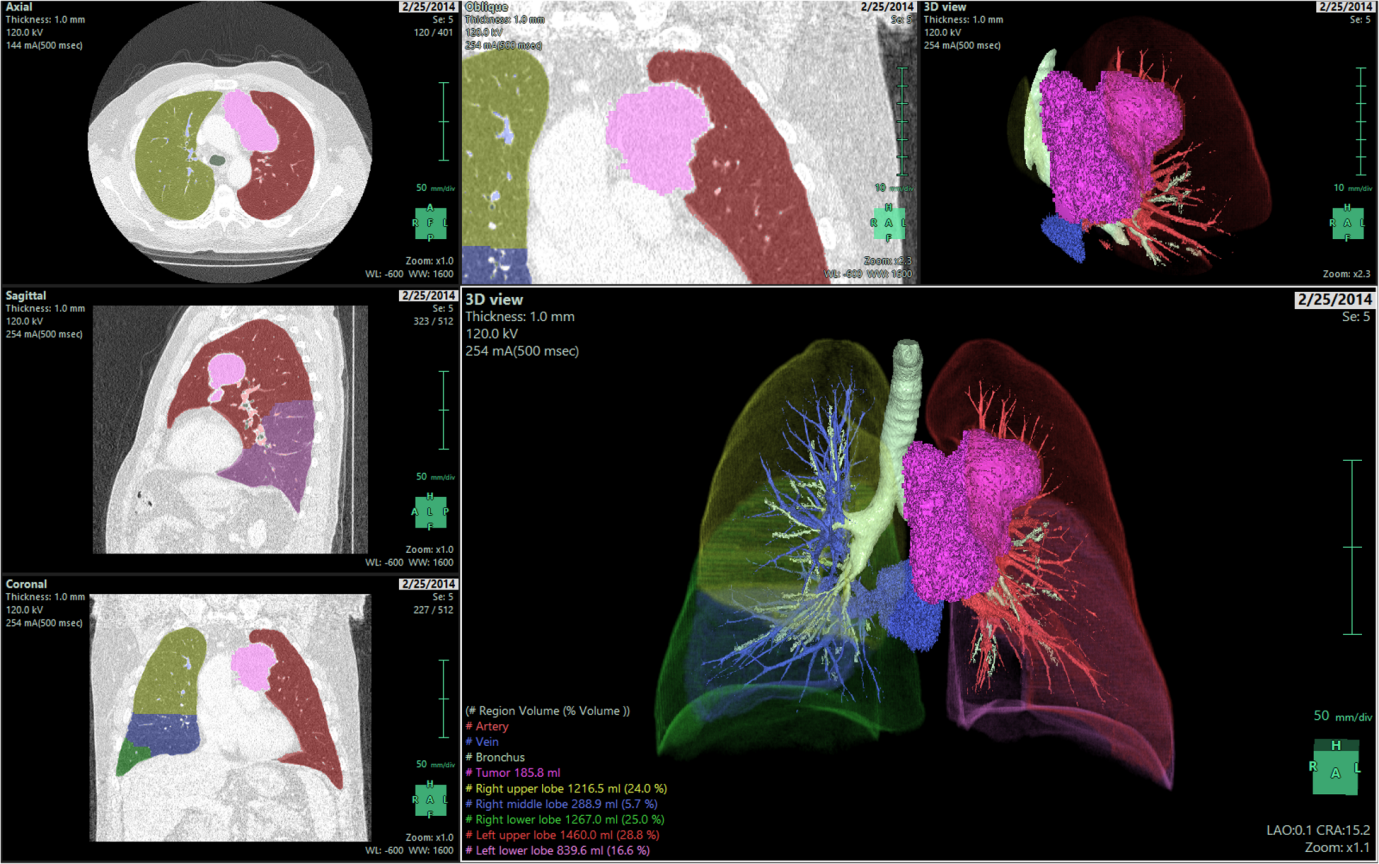
3D representation of regional segmentation of bronchus, artery, and vessels together with tumor volume

All patients underwent contrast-enhanced CT before biopsy and/or surgery to evaluate suspected mediastinal tumors. Chest CT examinations were performed with either 320-row detector CT (Toshiba Aquilion ONE, Otawara-shi, Japan), 64-row detector CT (Toshiba Aquilion 64, Otawara-shi, Japan), or 16-row detector CT (Siemens Somatom Sensation 16, Forcheim, Germany) scanners. The acquisition parameters were 0.5 mm, 0.5 mm, and 0.625 mm detector collimation; 120 kVp tube voltage; 0.5 s gantry rotation time; 1 mm, 1 mm, and 1.5 mm reconstructed section thickness; and 0.8 mm, 0.8 mm, and 1 mm reconstruction intervals. All examinations were performed after injecting 60–100 ml (1–1.5 ml/kg) of nonionic intravenous contrast agent (350/100 Omnipaque, GE healthcare, Oslo, Norway), at a rate of 2.5 ml/s via the antecubital vein. The area from the thoracic inlet caudally to include the adrenal glands was scanned. All images were reviewed by a senior radiologist (Ç.U) with more than 10 years of experience in thoracic imaging. She was blinded to the histopathological data to avoid bias. Multiplanar reformatted images were analyzed on a workstation (GE Healthcare, Waukesha, WI, USA) (Figs. 1 and 2).

### Patients and dataset management

Of the 83 patients included in analyses, 45 were male and 38 were female; the mean age was 49 ± 13.32 years (range 20–74 years). We used the Radcloud platform (Huiying Medical Technology Co., Ltd) to evaluate the data, and performed a radiomics statistical analysis. The training and validation datasets were separated by a random method 2:8 ratio with 502 random seeds. The histopathological diagnosis was noted from the pathology report.

### Image segmentation

Images were evaluated by two senior observers with 10 and 5 years of experience, respectively, in mediastinal surgery, and all disease lesions (VOIs) were annotated manually by observers who were blinded to the histopathological diagnoses of patients. Then, all images were re-evaluated by the senior radiologist. When there was ≥ 5% discrepancy, the radiologist made the final decision on the tumor borders. Because of the artifacts due to motion and breath during scan where the tumor margins could not be able to delineate precisely, 79 VOIs were included from the scans of the 83 patients and used for subject analysis to compute and extract the radiomics features.

### Feature extraction

In total, 1409 features were extracted from the CT images using the Radcloud platform. These features were classified into three groups. Table 2 lists the details of the groups: group 1 (first-order statistics-126 descriptors); group 2 (shape- and size-based features-14 features); group 3 (textural features-525 textural) (Table 2).

**Table 2:** Radiomics features selected for quantifying the heterogeneity differences

Radiomics group	Associated filter	Number of features	Radiomics features
First-order statistics	None	126	Energy, total energy, entropy, minimum, 10 percentile, 90 percentile, maximum, mean, median, interquartile range, range, mean absolute deviation, robust mean absolute deviation, root mean square, standard deviation, skewness, kurtosis, variance
Shape	None	14	Volume, surface area, surface volume ratio, spherical disproportion, maximum 3D diameter, maximum 2D diameter column, maximum 2D diameter row, elongation
Texture features	GLCM	525	Autocorrelation, average intensity, cluster prominence, cluster shade, cluster tendency, contrast, difference average, difference entropy, difference variance, dissimilarity, entropy, sum average, sum entropy, sum variance, sum squares
Texture features	GLSZM		Large area emphasis, gray level non-uniformity, size zone non-uniformity, gray-level variance, zone entropy, high gray-level zone emphasis, small area high gray-level emphasis, large area high gray-level emphasis
Texture features	GLRLM		Gray-level non-uniformity, run length non-uniformity, gray level variance, run entropy, high gray-level run emphasis, short run high gray-level emphasis, long run high level emphasis

*GLCM* gray-level co-occurrence matrix, *GLSZM* gray-level size zone matrix, *GLRLM* gray-level run length matrix

### Qualification

A large number of image features were measured. A dimensionality reduction was performed, and task-specific features were selected to identify the appropriate features. To reduce the redundant features, selection methods included the variance threshold (variance threshold = 0.8), SelectKBest, and the least absolute shrinkage and selection operator (LASSO), which were used to detect significant differences between low- and high-risk groups. Eigenvalues of the variance < 0.8 were removed. The SelectKBest method was used with a *p*-value to analyze the relationship between the features and the classification results. All features with *p*-values < 0.05 were used. The L1 regularizer was used as the cost function in the LASSO model; the error value of the cross-validation was 5, and the maximum number of iterations was 1000.

### Statistical analysis

Statistical analyses were performed in the Radcloud platform. The 1409 features identified were significantly correlated. The radiomics-based models were constructed with six classifiers: k-nearest neighbor (KNN), support vector machine (SVM), eXtreme Gradient Boosting (XGBoost), random forest (RF), logistic regression (LR), and decision tree (DT), and the validation method was used to improve the effectiveness of the model.

The following parameters were applied. For KNN: n_neighbors (5), weights (uniform). For SVM: kernel (rbf), C (1), gamma (auto), class_weight (balanced), decision_function_shape (ovr), random_state. For XGBoost: Eta (0.3), max_depth (6). For RF: n_estimators (10), class_weight (None). For LR: penalty (L2), C(1), solver (liblinear), class_weight (None), multi_class (ovr), random_state. For DT: splitter (best), criterion (gini).

The receiver operating characteristic (ROC) curve and the area under the curve (AUC) were used to assess the predictive performance of the training and validation datasets, respectively. The four indicators were P (precision = true positives/(true positives + false positives)), R (recall = true positives/(true positives + false negatives)), f1-score (f1-score = P × R × 2/(P + R)), and support (total number in test set) to evaluate the performance of the classifier.

## Results

Table 1 lists patient characteristics. The low-risk group included 51 patients (type A 10, type AB 19, type B1 22), and the high-risk group included 32 patients (type B2 14, type B3 18). No significant differences were observed between the low-risk and high-risk groups in either the training cohort (*n* = 66 cases) or the validation cohort (*n* = 17) (Table 1).

**Fig. 3:**
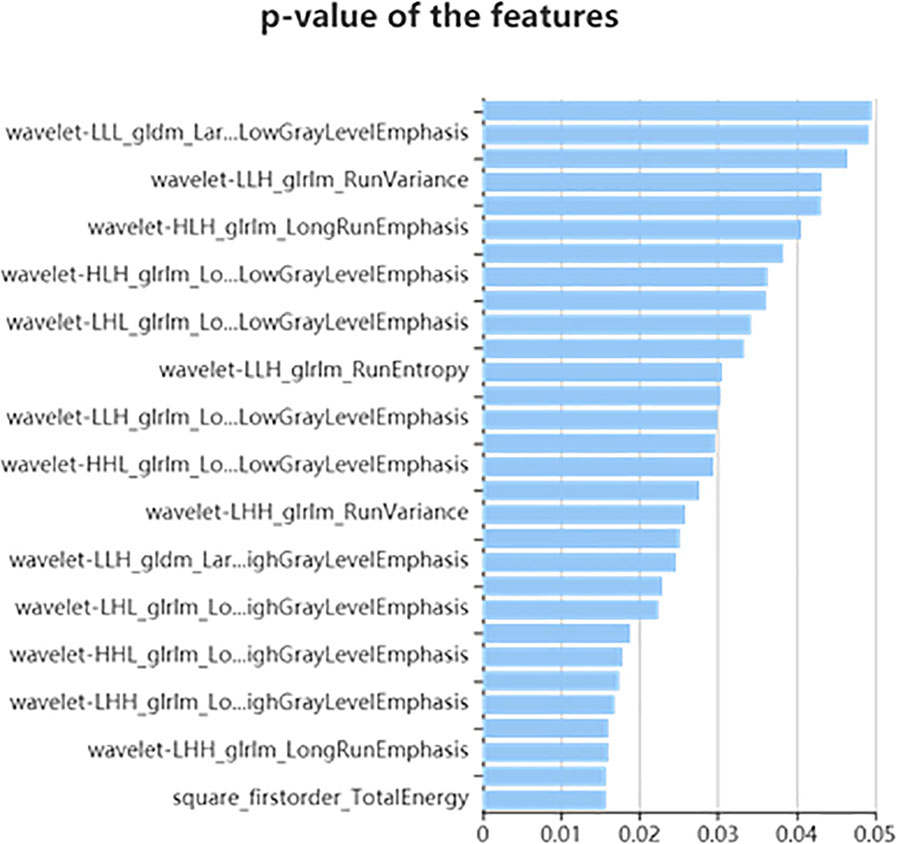
The SelectKBest method was used to further select the radiomics features; 30 features were selected

**Fig. 4:**
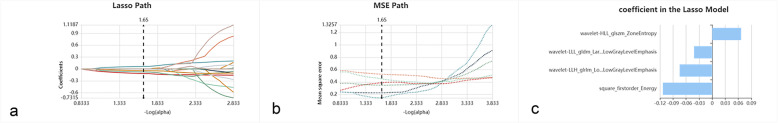
Lasso algorithm for feature selection. **a** Lasso path, **b** MSE path, **c** coefficients in the Lasso model. The Lasso model was used to select four features that correspond to the optimal alpha value

First, 459 features were selected from the 1409 total features using the variance threshold method, and 30 features were determined with the SelectKBest method (Fig. 3). Finally, four optimal features were defined with the Lasso algorithm (Fig. 4).

Figure 3 presents the ROC curve analysis results for the training and validation datasets to differentiate between the low-risk and high-risk groups of thymomas.

**Fig. 5:**
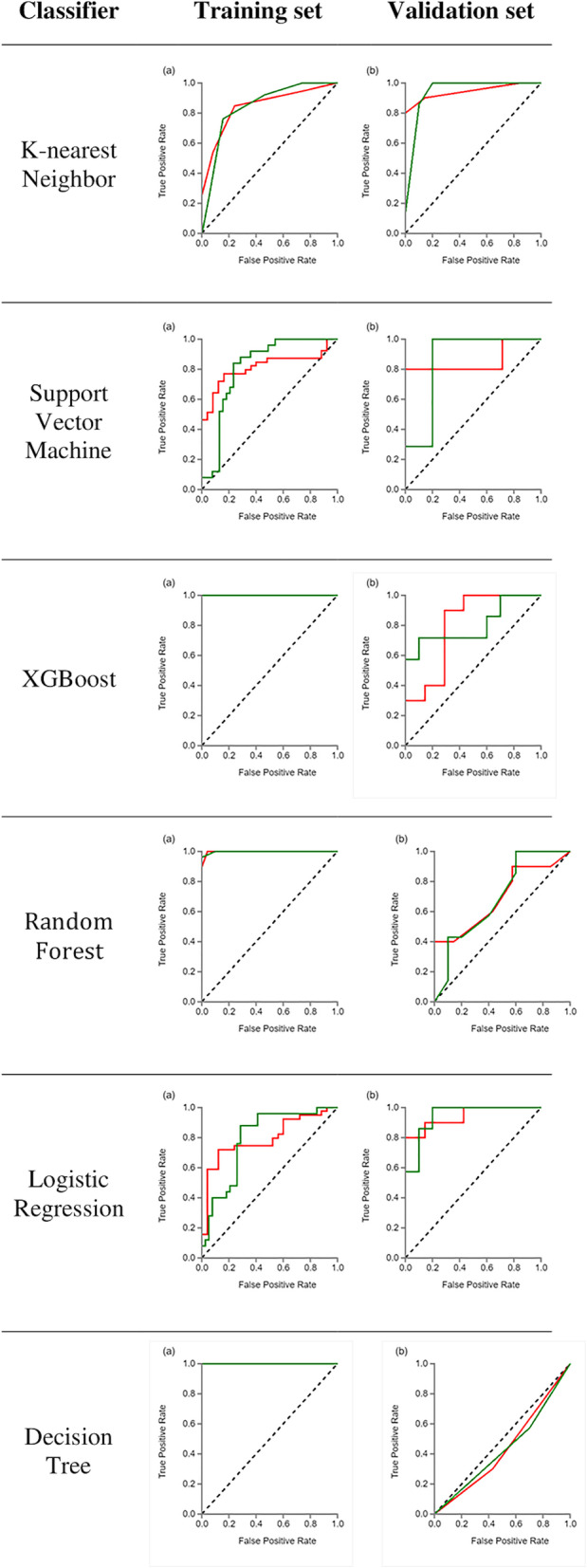
ROC curves of machine-learning methods for classification. Green indicates low-risk, and red indicates high-risk thymomas. **a** ROC curve of the training dataset, **b** ROC curve of the validation dataset

The AUC of XGBoost, RF, and DT machine learning methods was the highest at 0.998–1 for the training data while KNN and LR were highest for the validation data. Table 3 lists the results of the machine-learning classifiers of the validation set. The KNN scores were AUC = 0.943 for the low-risk group and AUC = 0.943 for the high-risk group. The LR scores were the same (AUC = 0.943) for both groups of thymomas. The KNN and LR classifiers were the best methods in the validation dataset in terms of differentiating between the low- and high-risk groups. Table 4 lists the diagnostic performance according to the four indicators. The ranges for the low-risk group were precision (0.5–0.9), recall (0.3–0.9), F1-score (0.37–0.9), and support (10), while the ranges for the high-risk group were precision (0.36–0.86), recall (0.57–0.86), F1-score (0.44–0.86), and support (7). The highest scores were achieved with the KNN machine-learning method (Fig. 5).

**Table 3:** ROC results with six machine-learning classifiers of validation set

Risk groups	Statistical measures	KNN	SVM	XGBoost	RF	LR	DT
Low	AUC	0.943	0.857	0.8	0.693	0.943	0.436
	95% CI	0.85–1	0.66–1	0.58–1	0.45–0.93	0.74–1	0.18–0.69
	Sensitivity	0.9	0.8	0.7	0.8	0.8	0.3
	Specificity	0.86	0.86	0.71	0.43	0.86	0.57
High	AUC	0.943	0.857	0.8	0.693	0.943	0.436
	95% CI	0.85–1	0.66–1	0.58–1	0.45–0.93	0.74–1.00	0.18–0.69
	Sensitivity	0.86	0.86	0.71	0.43	0.86	0.57
	Specificity	0.9	0.8	0.7	0.8	0.8	0.3

*KNN* k-nearest neighbor, *SVM* support vector machine, *XGBoost* eXtreme Gradient Boosting, *RF* random forest, *LR* logistic regression, *DT* decision tree

**Table 4:** The results of four indicators—precision, recall, F1-score, support in validation set

Risk groups	Indicators	KNN	SVM	XGBoost	RF	LR	DT
Low	Precision	0.9	0.89	0.78	0.67	0.89	0.5
	Recall	0.9	0.8	0.7	0.8	0.8	0.3
	F1-score	0.9	0.84	0.74	0.73	0.84	0.37
	Support	10	10	10	10	10	10
High	Precision	0.86	0.75	0.62	0.6	0.75	0.36
	Recall	0.86	0.86	0.71	0.43	0.86	0.57
	F1-score	0.86	0.8	0.67	0.5	0.8	0.44
	Support	7	7	7	7	7	7

*KNN* k-nearest neighbor, *SVM* support vector machine, *XGBoost* eXtreme Gradient Boosting, *RF* random forest, *LR* logistic regression, *DT* decision tree

Four radiomics features were identified that differentiated the low-risk group from the high-risk group of thymomas using machine learning, including Energy, Zone Entropy, Long Run Low Gray Level Emphasis, and Large Dependence Low Gray Level Emphasis (Fig. 4c).

Table 5 lists the details of the confusion matrix in the low-risk and high-risk thymoma groups using the best MLP learning classifier (KNN).

**Table 5:** The details of confusion matrix in low-risk and high-risk thymoma groups

KNN			
Risk groups	0	1	Accuracy (%)
Low	9	1	100
High	1	6	88
Accuracy (%)			94.3

*KNN* k-nearest neighbor

## Discussion

Radiomics has the potential to detect specific characteristics of a disease that cannot be visualized by current medical imaging modalities by quantitatively analyzing digital images. Recent studies have reported promising radiomics results in oncological practice. This method may supplement traditional imaging analysis and assist in providing personalized medicine for patients. Publications on applications of thoracic tumors have increased in recent years. In the present study, a radiomics platform was used to analyze both imaging and clinical data, and to perform a statistical analysis. Radiomics platforms have the potential to reveal distinct imaging algorithms that can be used to quantify the status of a disease, providing valuable knowledge for personalized medicine. They can also measure features in an imaging examination; shape, intensity, texture, wavelet, and Laplacian of Gaussian (*LoG)* features can be used to build predictive or prognostic non-invasive biomarkers or imaging modalities [18, 19].

This kind of platform can be used to extract radiomics features from two-dimensional (2D) and/or three dimensional (3D) images and dual masks on different imaging modalities, such as CT and MRI, which is why it was preferred for this study.

Thoracic oncology surgical information obtained from standard imaging modalities such as CT, MRI, and positron emission tomography scans usually refers to simple traits, such as gross shape, contrast enhancement, and size. However, imaging information is now much richer, and increased resolution quality has led to 3D image acquisitions containing millions of voxels available for analysis, making the development of radiomics a natural progression. Soon, data obtained from radiomics studies will be used to inform the diagnosis and treatment algorithms of thoracic malignancies.

Csutak et al. [18] in a recent study used textural analysis to quantify the fluid properties on computed tomography (CT) images of intraperitoneal effusions and evaluate its utility in differentiating benign from malignant collections. Similar to this textural studies, radiomics models have already been used to stage tumors and predict lymph node metastasis and prognosis [20–23]. A few studies have used radiomics models to predict the pathological invasiveness of TETs [24]. Although some previous studies have demonstrated that a textural analysis based on CT images can be used to differentiate high-risk TETs from low-risk TETs, they only analyzed 2D textural features, and their sample sizes were small [25, 26]. TETs are a heterogeneous group with different radiological appearances, histopathological features, and prognoses. They include thymomas, thymic carcinomas, and thymic neuroendocrine tumors, with a wide variety of histological features. Thymomas are the most common TETs and are subdivided into five groups (A, AB, B1, B2, and B3). These can resemble spindle cell tumors, lymphomas, or carcinomas depending on the tumor type. Interestingly, some recent studies have included thymic carcinomas as type C thymomas, which has not been used in the WHO classification since 2004, although thymic carcinomas have a heterogeneous morphology. Types A, AB, and B1 thymomas have a thymus-like architecture, whereas thymic carcinomas exhibit features that are encountered by other organs and are a heterogeneous group of tumors, such as squamous cell carcinoma, adenocarcinoma, and undifferentiated carcinoma [8]. For these reasons, thymic carcinomas and thymomas should not be analyzed together in a radiomics study.

CT and MRI are common imaging modalities to preoperatively assess thymomas. However, they have limited value for predicting the histological subtypes of TETs [14]. Jeong et al. reported that the contour of the tumor, mediastinal fat, and large vessel invasion are useful CT features to distinguish between the WHO classification subgroups [12]. CT and/or MR imaging findings can help differentiate between low-risk and high-risk thymomas among thymic carcinomas, but they are insufficient to distinguish between the different histological subtypes of the WHO thymoma classification.

Few studies have used a machine-learning system as an artificial intelligence approach with the application of radiomics features to analyze thymomas. This study addressed this research gap by developing a radiomics-based model incorporating machine learning to predict low- and high-risk thymomas.

Different machine-learning strategies, such as KNN, SVM, or RF decision trees, can be applied to construct the map of a given training set and a given set of features. During training, the parameters that define the mapping (whose representation depends on the chosen learning strategy) are iteratively refined such that estimation performance is maximized on the training set itself. Then, the difference between the given “ground truth” for each image and in the training set can be evaluated. The KNN classifier is a popular image classification algorithm that directly calculates image-to-image distances compared with other classifiers that need a training phase to calculate the distance between an image and a class [27]. RFs or random decision forests are ensemble learning methods for classification, regression, and other tasks that operate by constructing a multitude of decision trees at training time and outputting mode (classification) of the class or the mean prediction (regression) of the individual trees [28].

Wang et al. developed and compared the performance of radiomics signatures using textural features extracted from non-contrast-enhanced CT and contrast-enhanced CT scans [29]. They found that radiomics signatures performed better than radiologists with a high AUC, and that radiomics signatures based on a textural analysis extracted from a CT scan can be utilized as noninvasive biomarkers to differentiate high-risk thymomas from low-risk thymomas and advanced-stage thymomas from early-stage thymomas. They concluded that as a quantitative method, a radiomics signature provides complementary diagnostic information and informs plans for personalized treatment for patients with thymomas. Yang et al. studied a preoperative staging tool that differentiates Masaoka-Koga (MK) stage I patients from stage II patients using CT images of thymoma patients [30]. They used an artificial neural network (ANN) deep-learning model, namely, the 3D-DenseNet model, to distinguish the MK stage I and stage II thymomas. They found that deep learning has great potential to preoperatively stage thymomas, which dramatically improves identification between MK stage I and stage II thymomas compared with visual observations. They concluded that deep learning models can help guide surgical treatment and improve outcomes compared to traditional methods. Our findings are consistent with the previous results: a deep learning-supported radiomics model, such as an ANN, can help distinguish between low- and high-risk group thymomas. Similar findings have been reported elsewhere [31, 32]. A previous study detected correlations between preoperative CT imaging features and the biological behavior of thymomas [33]. Similarly, we found that myasthenia gravis, lactate dehydrogenase, and the largest tumor dimension size on CT (mm) were predictors of the prognosis. A previous retrospective study developed a radiomics model using LR analysis and realized high diagnostic performance [26]; it reported that the AUCs for differentiating high-risk thymomas from low-risk thymomas were 0.89 for mean0c and 0.87 for a combination of mean0u and entropy. Similarly, we found that the AUC of the radiomics signatures was 0.943 for KNN, the best MLP classifier.

The International Association for the Study of Lung Cancer and the International Thymic Malignancy Interest Group concluded that the WHO histological classification, the completeness of tumor resection, the MK stage, and the 8th edition of the TNM staging system are independent prognostic factors for TETs [34–36]. Similar relationships have been reported between thymomas and histological classification, completeness of tumor resection, and staging, but these relationships are not as strong as in some solid tumors, so the optimal staging system for TETs has not been defined. Therefore, histological classification and resectability are more useful determinants than staging systems, for both treatment decisions and predicting prognosis. An important element in thymoma surgical treatment planning is knowing the preoperative risk group, whether the thymoma is in the low- or high-risk group, may affect decisions about the surgical approach, which is one of the main determinants of the completeness of the resection. The low-risk group of thymomas is more likely to achieve complete resection with a MIS method; this may be less possible in the high-risk group. In the present study, we predicted thymoma risk groups by combining clinical and specific CT-based radiomics features with image variables, and this distinction may inform surgical treatment planning for thymomas. The most important advantage of this method is that it does not require a biopsy, which wastes time, and is costly, and can lead to complications.

Our study had some limitations. First, it was a retrospective study of thymomas from a single center, which may have caused selection bias. Second, it had a small sample size. A multicenter study with a larger sample size will be required to validate these results.

## Conclusion

The results of this study demonstrated that a machine**-**learning model and MLP classifier analysis can be used with CT images to predict low-risk and high-risk thymomas. The results also demonstrated that the combination of clinical and specific CT-based radiomics features and image variables can be used to predict thymoma risk groups. This method can be used as a preoperative technique to inform decisions about surgical approaches for treating thymoma.

## Data Availability

The datasets used and/or analyzed during the current study are available from the corresponding author on reasonable request.

## Abbreviations

ANN: Artificial neural network
AUC: Area under the curve
CT: Computed tomography
DT: Decision tree
IASLC: International Association for the Study of Lung Cancer
ITMIG: International Thymic Malignancy Interest Group
KNN: K-nearest neighbor
MG: Myasthenia gravis
MIS: Minimally invasive surgery
MK: Masaoka-Koga staging system
MLP: Multilayer perceptron
MRI: Magnetic resonance imaging
LASSO: Least absolute shrinkage and selection operator
LDH: Lactate dehydrogenase
*LoG*: Laplacian of Gaussian
LR: Logistic regression
P: Precision
PET: Positron emission tomography scan
R: Recall
RATS: Robotic-assisted thoracoscopic surgery
RF: Random forest
ROC: Receiver operating characteristic
SVM: Support vector machine
TETs: Thymic epithelial tumors
VATS: Video-assisted thoracoscopic surgery
*VOI*: Volume of interest
WHO: World Health Organization
XGBoost: eXtreme Gradient Boosting
2D: Two dimensional
3D: Three dimensional
